# Multiperspective and Multimethod Evaluation of Flexible and Integrative Psychiatric Care Models in Germany: Study Protocol of a Prospective, Controlled Multicenter Observational Study (PsychCare)

**DOI:** 10.3389/fpsyt.2021.659773

**Published:** 2021-06-01

**Authors:** Bettina Soltmann, Anne Neumann, Stefanie March, Ines Weinhold, Dennis Häckl, Roman Kliemt, Fabian Baum, Marcel Romanos, Julian Schwarz, Sebastian von Peter, Yuriy Ignatyev, Katrin Arnold, Enno Swart, Martin Heinze, Jochen Schmitt, Andrea Pfennig

**Affiliations:** ^1^Department of Psychiatry and Psychotherapy, Carl Gustav Carus University Hospital, Technische Universität Dresden, Dresden, Germany; ^2^Center of Evidence-Based Health Care, Medical Faculty Carl Gustav Carus, Carl Gustav Carus University Hospital, Technische Universität Dresden, Dresden, Germany; ^3^Department of Social Work, Health and Media, University of Applied Sciences Magdeburg-Stendal, Magdeburg, Germany; ^4^Institute of Social Medicine and Health Systems Research, Medical Faculty, Otto-von-Guericke-University Magdeburg, Magdeburg, Germany; ^5^WIG2 Scientific Institute for Health Economics and Health System Research Leipzig, Leipzig, Germany; ^6^Department of Child and Adolescent Psychiatry, Centre of Mental Health, Psychosomatics and Psychotherapy, University of Würzburg, Würzburg, Germany; ^7^Department of Psychiatry and Psychotherapy, Brandenburg Medical School Theodor Fontane, Immanuel Klinik Rüdersdorf, Rüdersdorf, Germany

**Keywords:** mental health care, cross-sectoral treatment, flexible and integrated treatment, FIT64b, mixed method approach

## Abstract

**Background:** New cross-sectoral mental health care models have been initiated in Germany to overcome the fragmentation of the German health care system. Starting in 2013, flexible and integrative psychiatric care model projects according to §64b SGB V German Social Law (FIT64b) have been implemented. The study “PsychCare” combines quantitative and qualitative primary data with routine health insurance data for the evaluation of these models. Effects, costs and cost-effectiveness from the perspectives of patients, relatives and care providers are compared with standard care. Additionally, quality indicators for a modern, flexible and integrated care are developed. This article describes the rationale, design and methods of the project.

**Methods:** “PsychCare” is built on a multiperspective and multimethod design. A controlled prospective multicenter cohort study is conducted with three data collection points (baseline assessment, follow-up after 9 and 15 months). A total of 18 hospitals (10 FIT64b model and 8 matched control hospitals) have consecutively recruited in- and outpatients with pre-specified common and/or severe psychiatric disorders. Primary endpoints are differences in change of health-related quality of life and treatment satisfaction. Sociodemographic and service receipt data of the primary data collection are linked with routine health insurance data. A cost-effectiveness analysis, a mixed method, participatory process evaluation by means of qualitative surveys and the development of quality indicators are further elements of “PsychCare.”

**Discussion and Practical Implications:** The results based on data from different methodological approaches will provide essential conclusions for the improvement of hospital based mental health care in Germany. This should result in the identification of key FIT64b elements that can be efficiently implemented into standard care in Germany and re-structure the care strongly aligned to patient needs.

**Clinical Trial Registration:** German Clinical Trial Register, identifier DRKS 00022535.

## Introduction

### Background

Mental disorders represent the leading causes of disability worldwide and result in a tremendous social and economic burden on individuals and health care systems (1–3). In Germany, recent epidemiological data indicate that almost 30% of the adult population (18–79 years) met the criteria for at least one mental disorder in the last 12 months and 13% of Germany's total health care budget is dedicated to mental health care (4, 5). In the last years, about 15–18 % of all registered days of absenteeism from work, especially long-term incapacity, were due to mental disorders (6, 7) and almost half of all newly approved applications for early retirements were attributable to mental disorders (8, 9). In addition to the socio-economic burden mental disorders interfere with adequate social functioning and permanently impair the quality of life of those affected. The severity, chronicity and high rate of comorbidity pose a challenge to the health care system and underline the need to focus particularly on improving the organization of the mental health care system.

Broad consensus exists that adequate contemporary mental health care comprises need-based, patient-centered, multiprofessional and cross-sectoral treatment. Therapeutic considerations and patient's needs and preferences should be decisive for the choice of setting since flexible treatment options ensure a consistent therapeutic relationship. Furthermore, the patients should be subjected to the least restrictive form of care (10–16).

However, this is often countered by a high degree of fragmentation of the health care system with rigid interfaces between sectors. In Germany, in- and outpatient services are separated on both the organizational and financial level. Furthermore, the fragmentation is reflected in the splitting of responsibilities across different social legislation for health insurance, rehabilitation, reintegration and care (17). Particularly for mental health care, sector boundaries are detrimental to patient care and cause problems like revolving door effects, poor information flow between service providers, and lack of cost-efficiency (18). The aforementioned facts points to the need for a reorganization of mental health care (19). Since the German health care reform in 2000, different approaches have been taken to set incentives for cross-sectoral care and new forms of integrated mental health care have been initiated legislatively, which have given new momentum to the field of mental health care (18–23).

§64b of the German social code book V (SGB V) introduced in 2012 has set the course for establishing flexible and integrated mental health care. FIT64b services are offered by hospital-based teams in contract with health insurers to overcome sector boundaries between in- and outpatient treatment within the hospital service provision. Common conceptual aims of the models include complex psychiatric treatment consisting of cross-sectoral care, shift of inpatient care to day-care and outpatient setting, improved transition between settings, efficient use of resources, patient-centered care, (personal) continuity and inclusion of social setting (13, 14, 17, 24). The reimbursement of the services in FIT64b projects is organized as a Global Treatment Budget (GTB), based on a capitation model agreed upon with the health insurance companies, irrespective of the chosen form of care and duration of treatment, as long as the number of people treated is within a corridor agreed between the negotiating parties (25). Currently, 22 individual model projects are being implemented in 10 German federal states. While almost half of the projects are based on former contracts (integrated care according to §140a SGB V or regional budgets; see Supplementary Material for a brief description of the corresponding laws.) others started out of standard care. Initially scheduled for a period of 8 years, the first models are due for renewal, which renders the obligation of evaluation even more crucial. Besides evaluations of precursor models -mainly dealing with regional budgets (25–34) and a monocentric evaluation (35), which, however, do not address cost-effectiveness, two model-spanning evaluations have been initiated: A controlled cohort study based on statutory health insurance claims data (EVA64) (36, 37) as well as an uncontrolled mixed-method patient and staff-oriented exploratory study (14, 38, 39). A multiperspective, multimethod, model-spanning approach including patient reported outcomes (PRO) to evaluate these models in comparison to standard care, however, was still missing. Further, so far, user perspectives have not been methodologically acknowledged for, as previous studies failed to use any participatory approach.

### Objective/Hypotheses

This project aims at comparing the effects, costs, cost-effectiveness and processes of a GTB according to §64b SGB V in the treatment of people with mental disorders compared to standard care. Our primary hypothesis is that patients treated within a model project show a higher improvement in quality of life and higher satisfaction with treatment compared to patients in standard care 15 months after inclusion into the study. It is moreover hypothesized that costs of care—including direct and indirect costs- for patients treated in model projects do not exceed those in standard care and that FIT64b models represent a cost-effective mental health care strategy to improve patients' health-related quality of life. In addition, the participatory process evaluation aims at (i) systematically evaluating the users' perspectives with FIT64b services, relating them to its grade of implementation and semi-quantitatively comparing them with standard care, and (ii) to analyzing the experiences of various stakeholder with FIT64b models. Another objective of the study is the development of quality indicators as these are highly relevant for cross-sectoral quality assurance and also regulated by law [§137a German social code book V (SGB V)].

## Methods

### Study Design

PsychCare is a controlled, prospective, multicenter cohort study conducted in 18 psychiatric hospitals in Germany and based on a multi-method approach combining quantitative and qualitative primary assessments, statutory health insurance (SHI) claims data and cost-effectiveness analysis. One part of the study is using a mixed method, participatory approach (40) or process evaluation (41) involving a collaborative research team of researchers with and without lived experiences (42), a multi-stakeholder analysis (43) and ethnographic methods (44, 45). Another part is dedicated to the development of quality indicators for a flexible and integrated mental health care.

#### Model Hospitals

All hospitals that have concluded a contract with at least one SHI fund in accordance with §64b SGB V were eligible to be included into the stratified selection. Two strata were formed, based on whether the contract was concluded before or after 2015 to distinguish between models with at least 4 years of experience and newly established care models.

#### Control Hospitals

Hospitals with a psychiatric in- and outpatient unit (“PIA” psychiatric institute ambulance) and without a contract according to §64b SGB V were eligible to be included into the study.

#### Matching of Hospitals

Model and control hospitals were matched according to an algorithm developed by one of the project partners (36, 46). Briefly, data from structured quality hospital reports (SQR) and the German spatial sociodemographic and socioeconomic database INKAR (Indicators and Maps for Spatial and Urban Development) were used (47). Since 2005, hospitals in Germany are legally obliged (§137 SGB V) to publish structured quality reports every 2 years containing data on structure, processes and performance of each hospital department (48). INKAR data of corresponding administrative districts are publicly available and provided by the German Federal Office for Building, and Regional Planning (BBSR). Three different types of criteria were developed for the selection of suitable control hospitals: mandatory criteria (institutional structure of a specialized Department of Psychiatry and Psychotherapy, existence of a psychiatric institutional outpatient department (PIA), same regional Association of Statutory Health Insurance Physicians), criteria based on patients' characteristics (i.e., number of cases per mental health diagnosis) and structural characteristics of the hospitals (i.e., number of beds) and the environment (rate of unemployment, mean household income, number of physicians per inhabitants) which were used with a pre-specified weighting for the selection of control clinics.

In each German federal state with a selected model hospital, the first 10 best matching control hospitals were asked to participate in the study successively until a consenting hospital was found.

### Procedure and Setting

From February 2018 until September 2019 in- and outpatients of the participating hospitals were consecutively screened for the inclusion and exclusion criteria (see below), informed about the aim and procedure of the study and asked for willingness to participate. Patients that consented to take part in the quantitative assessments were asked to name a relative or another individual living with them or providing support who then was asked for willingness to participate. Additionally, participants were asked to consent for using health insurance claims data and linking it with primary data. Written informed consent was obtained from patients and relatives. In the case of underage patients, parents or other custodians were also asked for their consent. The participatory process evaluation uses a mixed method approach, involving routine data from the study centers, qualitative interviews, two different standardized surveys, ethnographic methods and a multi-stakeholder analysis; patients taking part in this study part were separately asked for consent.

### Study Population

#### Inclusion Criteria

1) In- or outpatient treatment in one of the participating hospitals during recruitment phase
2) Meeting one of the following combinations of clinical diagnosis according to ICD-10 (49) at admission and verified at discharge and age (subgroups)
  a) mental and behavioral disorders due to use of alcohol (F10) and age of ≥ 18 years
  b) mood affective disorder (F30-F39) and age of ≥ 18 years
  c) schizophrenia, schizotypal disorder, delusional disorder or brief psychotic disorder (F20-23) and age of ≥ 18 years
  d) behavioral and emotional disorder (F90-98) and age of 6–17 years
  e) eating disorder (F50) and age of 12–25 years
  f) mental and behavioral disorder due to use of alcohol (F10) and age of 12–17 years
  g) mood affective disorder and age of 6–17 years
  h) schizophrenia, schizotypal disorder, delusional disorder or brief psychotic disorder (F20–23) and age of 12–17 years
3) Sufficient command of German to take part in the study
4) Capacity to provide informed consent

#### Exclusion Criteria

1) Severe organic brain dysfunction including impairment of cognitive function
2) Severe intellectual disabilities
3) Acute suicidality

### Outcome Assessments

#### Quantitative Assessment of Patient-Reported Outcomes

The quantitative assessments are administered longitudinally at three data collection points (baseline, follow-up after 9 and after 15 months).

##### Primary Outcomes

The primary outcomes are differences in

- health-related quality of life
- treatment satisfaction

from baseline to month 15 between model and control group. The primary outcome is calculated separately for eight subgroups determined by diagnosis and age {cf. inclusion criteria [2 (a)–(h)]}.

##### Secondary Outcomes

Differences from baseline to month 9 and 15 between model and control group

Patients:

- subjective burden of psychiatric symptoms
- occupational integration (age ≥ 18), days of absence from school (age <18 years)
- recovery
- involvement in and satisfaction with clinical decision making
- direct and indirect mental health care costs

Relatives:

- experience of burden of family care givers
- satisfaction of family care givers with support

Instruments are listed in Table 1.

**Table 1:** Overview of instruments to quantitatively assess patient-reported outcomes.

**Outcome**	**Instrument**	**Group**	**Reference**
health-related quality of life	Quality of well-being self-administered scale (QWB-SA)	Patients ≥18	(50)
	KIDSCREEN	Patients <18	(51)
treatment satisfaction	ZUF-8	All patients	(52)
subjective burden of psychiatric symptoms	Symptom Checklist (SCL-9)	Patients ≥18	(53)
	Strengths and Difficulties Questionnaire (SDQ)	Patients <18	(54)
recovery	RAS-R	All patients	(55)
involvement in and satisfaction with clinical decision making	CDRC-P, CDIS-P		(56, 57)
recording of costs for mental health care	CSSRI		(58)
experience of burden of family care givers	Questionnaire on the burden on relatives (FBA)	Relatives	(59)
satisfaction of family care givers with support	EUFAMI Part B (European Federation of Associations of Families of Mentally Ill People)		(60)

##### Linkage of Primary and SHI Data

**Figure 1:**
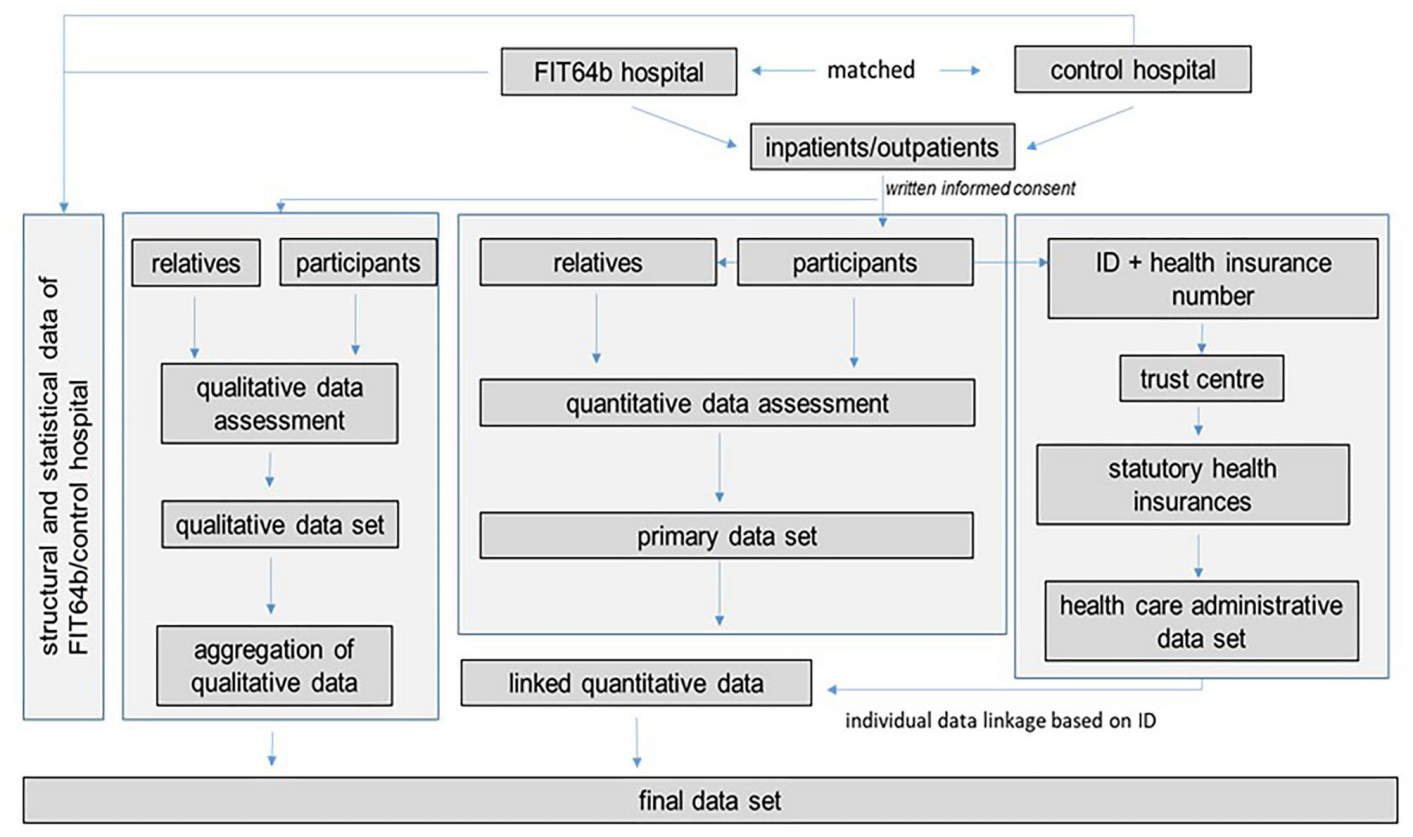
Study flow chart.

An individual data linkage of the primary quantitative data and claims data of SHI is conducted. Individual health insurance numbers are collected by an independent trust center, sorted and corresponding data requested from the participating health insurance funds. SHI data include basic patient related sociodemographic and morbidity data (e.g., age, sex, disability), sick leave, use of inpatient and outpatient services, pharmaceutical and non-pharmaceutical treatment. Claims structure and contents correspondent to that of the EVA64 study (36). Pseudonymised data are transferred to the research unit and linked with primary data by study identification number (see Figure 1). The procedure is in line with Good Practice in Secondary Data Analysis and Reporting (61, 62) as well as Good Practice Data Linkage (63).

#### Cost-Effectiveness Analysis

##### Costs

The study includes an economic evaluation. Health care utilization is captured based on an adapted and piloted version of the German Client Service Receipt Inventory (CSSRI) (57). Direct medical, direct nonmedical and indirect costs are then derived by combining these utilization data with appropriate unit costs. The data acquisition includes the following categories:

- direct medical costs: inpatient treatment days, inpatient-equivalent treatment days, day care/outpatient attendances, rehabilitation services, primary care contacts, community-based service contacts, psychosocial interventions, pharmaceuticals, remedies and aids
- direct non-medical costs: non-physician health services, social services, supported accommodation usage, domestic help
- indirect costs: loss of income due to sick leave, early retirement due to sickness, time and expenditure of relatives, premature death

The recorded types of utilized services are priced by using negotiated prices from SHI routine data and aggregated data for staff, material and infrastructure expenses from hospital information and empirical valuation rates according to Grupp et al. (64) for complementary psychiatric services and Krauth et al. (65) for inpatient somatic care, medical rehabilitation and SHI-accredited physicians. The individual data linkage (Figure 1) furthermore enables resource utilization determined via the primary data collection to be validated by corresponding health care utilization determined from SHI claims data.

##### Cost-Effectiveness

Cost-effectiveness of FIT64bmodel care is evaluated based on the incremental cost-effectiveness-ratio (ICER) measuring the incremental cost per quality-adjusted life year (QALY) gained. QALYs will be calculated based on health utilities extracted from the QWB-SA (50). The calculation of the ICER will include direct and indirect medical and non-medical costs, thus taking a societal evaluation perspective.

#### Participatory Process Evaluation

Implementation of FIT64b service elements comprises multiple interacting components on several organizational levels. To evaluate these complex interventions, a participatory-collaborative approach was chosen involving researchers with and without own experiential expertise with the mental health care system, as well as a multi-stakeholder approach including staff from statutory health insurances and hospitals (medical and economic controlling, management and senior physicians).

A mixed method approach is used for this process evaluation to acknowledge the different perspectives of various stakeholders of FIT64b models and to be able to triangulate and validate results (41). The method of process evaluation is described below.

Participatory, mixed method evaluation of the service users' experiences: The SEPICC questionnaire (38) and a specific set of quantified program components are used (24) that have been developed in a precursor study, assessing the processual and structural aspects of FIT64b models (14). Another set of FIT64b specific program components and a second questionnaire is being developed in the current study that both aim at evaluating the services users' experience, deliberately making use of the experiential expertise of the researchers with lived experiences, that is, with personel experience of the psychiatric care system, mental crisis and recovery, on the level of the research team. Semi-structured in-depth interviews with services users are added, using coding tandems, each one consisting of a researcher with and without lived experiences, and following principles of the Grounded Theory Methodology (66) and the Qualitative Comparative Analysis (67). Interviews are audio-taped and transcribed verbatim ensuring the removal of any identifying information to maintain anonymity and confidentiality. The transcripts are coded and analyzed by computer assisted qualitative data analysis (NViVo-Software).

Multi-stakeholder approach: As a preceding study researching the implementation of GTB in Germany could identify potential obstacles to implementation and diffusion of GTB (16), the present study included a qualitative multi-stakeholder approach to a) explore the incentives, requirements and challenges of this form of hospital reimbursement and b) describe the process of implementation from an organizational perspective. This includes focus groups and expert interviews (68) with management and controlling staff from FIT64b adopting hospitals and payers (e.g., corresponding health insurances). The collected data is processed using qualitative content analysis (69) to summarize the material and to unfold further details about implementation of GTB and the dissemination of FIT64b.

Ethnographic approach: An additional ethnographic approach, including participant observation, in three model and control hospitals contributes (a) to evaluate the changes of the everyday routines of staff following the FIT64b structural alterations and (b) to analytically associate the modifications on the organizational and structural levels with interpretations and meanings on the individual level in practice (70–72).

#### Development of Quality Indicators

This part of the study aims to develop quality indicators for the short- and medium-term monitoring of flexible and integrated mental health care. The quality indicators focus on quality domains derived from the objectives of the FIT64b models: coordination, cooperation, continuity; patient orientation; access and availability to outpatient or day hospital care. In addition, items developed by patient-experts on experience-measures have been developed. The set of indicators is developed by means of an iterative process using evidence synthesis and consensus processes.

The evidence search was conducted in (1) the databases QUINTH (quality indicator thesaurus of the Statutory Health Insurance Funds Association) and NQMC (national quality measures clearinghouses), (2) German evidence and consensus based (S3-) guidelines, (3) Medline. Retrieved quality indicators are extracted as quality aspects in order to group similar aspects with different operationalizations.

The formal consensus process follows a RAND/UCLA Appropriateness Method (RAM), a modified Delphi technique based on a multi-stage formal consensus procedure (73). Members of the expert panel were selected based on expertise in mental health care and included psychiatrists, experts in mental health service research, patient and relative representatives of national organizations and further experts in the field including patients and relatives. The final expert panel consisted of 15 representatives of national organizations, 10 experts, 12 patients/relatives of national organizations and 12 independent patients/relatives. Three Delphi-rounds are conducted. In addition to rating relevance and influenceability, panel members could comment on each quality aspect. Rating is conducted by postal mail or e-mail as preferred by the panel member. Rating of relevance and influenceability is processed on a 5-point Likert scale (1 = strongly disagree; 2 = disagree 3 = partly agree/disagree; 4 = agree; 5 = strongly agree). Comments are categorized, hierarchised and, if necessary, feedbacked in the subsequent round. An aspect was defined “consented” if at least 75 % of all panel members agreed or strongly agreed that the aspect was relevant and influenceable in the second round of the individual quality aspect. The consented set of aspects is operationalised by the project group according to the specifications developed by the healthcare quality assurance organization (IQTIG) (74).

The consented set of quality indicators will be piloted in one model and one control hospital. After revision, the set will be finalized.

### Statistical Analyses

The primary outcomes are changes in quality of life and satisfaction with care over 15 months between groups. Sample size calculation was based on satisfaction with care. To detect an effect size of 0.39 with a significance level of 0.05, a power of 0.8 and an expected loss-to-follow up of 25%, a sample of 110 patients per diagnostic subgroup a)-c) (see section Inclusion Criteria) is necessary, that is, 330 patients each in model and control hospitals (75).

Statistical analysis will employ generalized linear effects models accounting for the patient-wise error in the repeated measures design. The main analysis will focus on group differences in intervention and control group regarding primary and secondary outcomes. On the level of hospitals, more in-depths analysis will include stratification between model hospitals that already incorporated model-like structures prior to the onset of FIT64b and those that started from scratch. On the patient level, further stratification will focus on identifying differential effects within distinct sub groups of inclusion diagnoses (see section Inclusion Criteria bullet point 2) and distinguish effects between patients with a relatively short vs. longer history of treatment. The primary analysis will be based on the ITT sample. This means that patients will be considered in the model (or control) group, if they were treated within a FIT64b model (or control hospital) regardless of the interventions delivered and independent from whether they change hospital.

Fidelity to the FIT64b model is measured in model and control hospitals using 11 previously developed and empirically based components that comprise treatment structures and processes (14, 24, 39).

Cost differences between model and control group will be evaluated by comparison of the mean cost values calculated individually for each insured person. Comparison will be based on a suitable two-sided test for independent random samples. Since in the present panel structure (for each individual there is one observation for each observed period) the linear model assumption of independence of observations could be violated, cluster-robust standard errors are used to assess the statistical significance of the results. Furthermore, variances in cost developments are explained by using multivariate difference-in-difference regression models.

## Trial Status Phase

Ten out of 17 eligible FIT64b model hospitals and eight matched control hospitals consented to take part in the study. Quantitative baseline and qualitative data collection lasted from March 2018 to September 2019. The last follow-up is completed by the end of December 2020. SHI data were transferred at two time points (December 31, 2019 and December 31, 2020) and cover data for the period of two years prior to baseline assessment until the end of 2019. Data collection took partly place during the COVID-19 pandemic. However, baseline assessment, which required on-site recruitment, was completed in September 2019. The follow-up procedure could be conducted as planned via postal follow-up survey.

## Discussion

This multiperspective and multimethod evaluation study compares the effects, costs and cost-effectiveness and processes of a global treatment budget (GTB) according to the German social law §64b SGB V in the treatment of people with mental disorders compared to standard care in Germany. The results will provide essential evidence of which form of cross-sectoral mental health care is associated with better outcomes. Effective elements of FIT64b models will be identified that can be implemented into standard care, thus fostering the optimization and re-structuring of mental health care. Cost-effectiveness analyses will examine whether FIT64b models are cost-effective mental health care strategies to improve health-related quality of life of psychiatric patients.

### Strengths

This is the first study to combine a multiperspective and multimethod approach and a controlled design to evaluate cross-sectoral mental health care in a FIT64b-model-overarching sample in Germany. PsychCare is based on (1) patient-reported outcomes, (2) participatory process evaluation, (3) use of resources, (4) cost-effectiveness, (5) development of quality indicators, and (6) integration of data (quantitative and qualitative data; primary and secondary data).

One major strength of this study is the multimethod approach which attempts to overcome the limitations of previous studies focusing on only one data source (24, 36). To the best of our knowledge, this study is the first to capture and link primary data on service use and claims data in individuals with mental disorders in Germany, thus enabling cross-validation. Concerns regarding the accuracy of self-reports have been raised especially for mental health populations (76). The precision of self-reported data on health care utilization is mainly affected by cognitive abilities, recall time frame, type of service utilization and frequency (77). On the other hand, administrative data cover only the payer perspective, i. e. billed services and associated information on for example diagnoses and prescribed medication. The service user is the only person accumulating complete information about which formal and informal health services have been received. An accurate acquisition of service use and cost data is an indispensable prerequisite to evaluate the cost-effectiveness of health care models (78).

Following a convergent parallel design patient-reported outcomes are collected both quantitatively and qualitatively, allowing representative results to be gained and patient-relevant aspects to be identified for an overall interpretation.

The presented evaluation takes the perspective of patients, relatives and care providers into account. Although the main objective of FIT64b models is to obtain better patient-related outcomes, the perspectives of relatives and care providers have to be taken into account, regarding workload, efficient use of resources, optimization of organizational processes etc. As new forms of health care provision have to be feasible and acceptable for providers, the involvement of multiple perspectives guarantees a broad acceptance and improved implementation of new health care provision models. Need perception may differ considerably between professionals and patients and disagreement may exist concerning unmet needs. This multiperspective approach provides the opportunity to identify potential areas of discrepancy and offer a more comprehensive view.

All model projects had already concluded §64b contracts 2 to 5 years before the start of recruitment. Thus, model hospitals have had already established FIT64b care approaches. Consequently, novelty bias is likely to be neglectable (79).

The evaluation is assessed under real world conditions assuming a high external validity of the results (80). The study design is controlled; we have no evidence that the COVID-19 pandemic situation should affect the groups being compared differently.

### Limitations

We are conscious of the following limitations of the study. The FIT64b care models implemented in the different model hospitals are heterogeneous. Some evolved from precursor projects/contracts like regional mental health care budget or selective contracting within integrated care projects. As it is within the conceptual framework of the model to flexibly adapt care elements to patients' needs, variations between individual models are inbuilt. Furthermore, the included FIT64b models have contracts with different numbers of SHI funds, from single contracting to regional budgets including all SHI funds.

We are aware of selection bias due to consent to enroll in the study, both on the hospital and patient side. However, each control hospital is within the top 10 matching control hospitals and both the model and control hospitals reflect a wide range of geographical areas within Germany. The matching algorithm is based on selected a priori defined criteria combining the hospitals' services and patients. These criteria do not fully represent patient characteristics beyond mental health disorders such as ethnicity, country of origin or LGBT status. Conclusions if FIT64b works better for subgroups facing disparities in standard care might be limited.

Selection bias toward patients who are less severely ill and therefore more willing and able to participate cannot be ruled out completely. However, since all patients were consecutively screened the bias was reduced to the lowest possible level. Reasons for being not-eligible or not willing to participate are documented. The results of this study based on data from different methodological approaches and perspectives will provide essential conclusions for the optimization of mental health care. This will result in potential re-structuring of care strongly aligned to patient needs and identification of elements of integrated and continuous care that can be efficiently implemented into standard care in Germany.

## Ethics Statement

The studies involving human participants were reviewed and approved by Institutional Review Board (IRB00001473 and IORG0001076) of the Medical Faculty of the Technical University Dresden and at each site where a separate approval was mandatory. The participants provided their written informed consent to participate in this study. In the case of minors, written informed consent was provided by the parents/legal guardians and assent was obtained from the underage patient.

## Author Contributions

BS, AN, SM, IW, DH, RK, FB, MR, JuS, SP, YI, KA, ES, MH, JoS, and AP contributed to the design and study protocol. BS and AP drafted the manuscript. AN, SM, IW, DH, RK, FB, MR, JuS, SP, YI, KA, ES, MH, and JoS critically revised the manuscript. All authors approved the final version.

## Conflict of Interest

The authors declare that the research was conducted in the absence of any commercial or financial relationships that could be construed as a potential conflict of interest.

## Abbreviations

BBSR, German Federal Office for Building: and Regional Planning
CSSRI: Client Service Receipt Inventory
FIT64b: flexible and integrative psychiatric care model project according to §64b SGB V
GTB: Global Treatment Budget
ICER: incremental cost-effectiveness-ratio
INKAR: Indicators and Maps for Spatial and Urban Development
IQTIG: healthcare quality assurance organization
NQMC: national quality measures clearinghouses
PIA: psychiatric institute ambulance
PRO: patient reported outcomes
QALY: quality-adjusted life year
QUINTH: quality indicator thesaurus of the Statutory Health Insurance Funds Association
SGB: German Social Code Book (Sozialgesetzbuch)
SHI: statutory health insurance
SQR: structured quality hospital reports.
