# Supplementary material for: Multiperspective and Multimethod Evaluation of Flexible and Integrative Psychiatric Care Models in Germany: Study Protocol of a Prospective, Controlled Multicenter Observational Study (PsychCare)
Source: Front Psychiatry. 2021 Jun 1;12:659773. doi: 10.3389/fpsyt.2021.659773 (PMC8205541; doi:10.3389/fpsyt.2021.659773)
Supplement: Supplementary file 1 [file Table_1.docx]

Supplementary material

*Brief description of the laws and their influence on integrated care and the forms of contracting in mental health care in Germany mentioned in the article (1)*

-**German Health Care Reform Act** (“GKV^[[1]](#footnote-1)^-Gesundheitsreformgesetz”)

In the course of the German Health Care Reform Act in 2000, the legislative authorities allowed several innovative models of care by providing for the first time the legal basis for integrated care (§140 of the fifth German Social Code Book (SGB V)), as a new component of standard care. Among these are the so called **Integrated Care Models**, the Nationwide Disease Management programs and the Regional Psychiatry Budget. The mandatory involvement of the Federal Association of Statutory Health Insurance Physicians and the complicated reimbursement mechanisms, led to only a halting development of integrated care. This form of care was decisively developed further in 2004 with the Health Care Modernization Act.

-**Health Care Modernization Act** (“GKV^1^-Modernisierungsgesetz”)

Since 2004, individual physicians and physician’s networks have been able to become direct contract partners with the health insurances for the first time through selective contracts (2). A start-up financing was introduced, statutory health insurances could withhold up to 1% of the total reimbursement from in- and outpatient care to foster integrated care. By the end of 2007, 5,265 integrated care contracts had been registered with all medical specialities, of which only 77 were related to mental disorders (3).

-**Regional Psychiatry Budget** (RPB)

The RPB (according to §26 Federal Hospital refund Regulation) represents a special arrangement within the German Federal Hospital Refund Regulation, based on the capitation principle. A lump sum is allocated to a major inpatient care provider in a defined region on a yearly basis. The provider is free to offer all forms of treatment and to construct individual models of integrated care that specifically suit the region and the needs of the community members. The provider does not need to itemise services and will not be supervised by the medical review board of the statutory health insurane companies (4).

**- flexible and integrative psychiatric care model projects according to §64b SGB V German Social Law (FIT64b)**

The German social law §64b SGB V launched in 2012 enables statutory health insurance funds, together with hospitals, to jointly establish contracts based on a global treatment budget (GTB) covering costs for all psychiatric hospital services and is related to the number of patients treated. The GTB covers in-, outpatient and day care with the opportunity to apply an individual treatment strategy in order to provide continious, flexible and integrated treatment (5). The statutory health insurances are obliged to arrange a scientific evaluation of the projects by independent experts.

REFERENCES

1. Milstein R, Blankart CR. The Health Care Strengthening Act: The next level of integrated care in Germany. *Health Policy* (2016) **120**:445–51. doi:10.1016/j.healthpol.2016.04.006

2. Amelung V, Hildebrandt H, Wolf S. Integrated care in Germany-a stony but necessary road! *Int J Integr Care* (2012) **12**:e16. doi:10.5334/ijic.853

3. Bröcheler A, Bergmann F, Schneider F. Models of mental health care in psychiatry across sectoral borders. *Eur Arch Psychiatry Clin Neurosci* (2009) **259 Suppl 2**:S227-32. doi:10.1007/s00406-009-0054-9

4. Berghöfer A, Hubmann S, Birker T, Hejnal T, Fischer F. Evaluation of Quality Indicators of Integrated Care in a Regional Psychiatry Budget - A Pre-Post Comparison by Secondary Data Analysis. *Int J Integr Care* (2016) **16**:17. doi:10.5334/ijic.2479

5. Baum F, Schoffer O, Neumann A, Seifert M, Kliemt R, March S, et al. Effectiveness of Global Treatment Budgets for Patients With Mental Disorders-Claims Data Based Meta-Analysis of 13 Controlled Studies From Germany. *Front Psychiatry* (2020) **11**:131. doi:10.3389/fpsyt.2020.00131

1. GKV= statutory health insurance (SHI) [↑](#footnote-ref-1)
